# *Wolbachia*-Induced aae-miR-12 miRNA Negatively Regulates the Expression of *MCT1* and *MCM6* Genes in *Wolbachia*-Infected Mosquito Cell Line

**DOI:** 10.1371/journal.pone.0050049

**Published:** 2012-11-16

**Authors:** Solomon Osei-Amo, Mazhar Hussain, Scott L. O’Neill, Sassan Asgari

**Affiliations:** 1 School of Biological Sciences, The University of Queensland, Brisbane, Queensland, Australia; 2 School of Biological Sciences, Monash University, Clayton, Victoria, Australia; University Of Montana - Missoula, United States of America

## Abstract

**Background:**

Best recognized for its role in manipulating host reproduction, the parasitic gram-negative *Wolbachia pipientis* is known to colonize a wide range of invertebrates. The endosymbiotic bacterium has recently been shown to cause a life-shortening effect as well as inhibiting replication of arboviruses in *Aades aegypti*; although the molecular mechanisms behind these effects are largely unknown. MicroRNAs (miRNAs) have been determined to have a wide range of roles in regulating gene expression in eukaryotes. A recent study showed that several *A. aegypti* mosquito miRNAs are differentially expressed when infected with *Wolbachia.*

**Methodology/Principal Findings:**

Based on the prior knowledge that one of these miRNAs, aae-miR-12, is differentially expressed in mosquitoes infected with *Wolbachia,* we aimed to determine any significance of this mediation. We also set out to characterize the target genes of this miRNA in the *A. aegpyti* genome. Bioinformatic approaches predicted a list of potential target genes and subsequent functional analyses confirmed that two of these, DNA replication licensing (*MCM6*) and monocarboxylate transporter (*MCT1*), are under the regulative control of aae-miR-12. We also demonstrated that aae-miR-12 is critical in the persistence of *Wolbachia* in the host cell.

**Conclusions/Significance:**

Our study has identified two target genes of aae-miR-12, a differentially expressed mosquito miRNA in *Wolbachia*-infected cells, and determined that the miRNA affects *Wolbachia* density in the host cells.

## Introduction

*Wolbachia* is an endosymbiotic gram-negative bacterium which is estimated to infect as many as two thirds of all insect species [1]. This often parasitic endosymbiont is most commonly associated with manipulating host reproductive strategies through means such as feminization and cytoplasmic incompatibility [2]; although some strains of the bacterium have been known to have mutualistic or commensal interactions with their hosts [3].

In 2008, McMeniman et al. introduced the *w*MelPop-CLA strain of *Wolbachia* into *Aedes aegypti*, which is the main vector of dengue viruses. This had the effect of reducing the adult female’s lifespan by as much as 50% [4]. In addition to the life-shortening effect, *Wolbachia* infection of *A. aegypti* was shown to inhibit replication of several pathogens, including dengue viruses [5]. Despite the significance of these findings, relatively little is known about the molecular mechanisms which mediate the changes made to the mosquito’s biology upon infection with *Wolbachia*.

Since their discovery in 1993 [6], microRNAs (miRNAs), as ∼22 nucleotide non-coding RNAs, have been credited with an ever-expanding range of roles in regulating expression of genes. Seemingly specific to eukaryotes, their initial discovery in round worms illustrated the role of miRNAs in post-transcriptional control over genes responsible for developmental timing [7]. Since then, miRNAs have been implicated in many more biological processes [8]. There are multiple different modes of miRNA actions currently described which rely on the interaction of miRNAs with their target mRNAs [9]. Binding of a miRNA to its target mRNA may lead to repression of translation, degradation of target transcripts or even up-regulation of transcript levels [10], [11].

MiRNAs can also play a part in interactions between organisms. One such scenario was described in the case of *Helicobacter pylori.* It was demonstrated by Fehri et al. (2010) that infection with *H. pylori* induces the expression of miR-155 and down-regulates miR-218 [12]. This overall manipulation of host miRNAs results in an alteration to metabolic pathways within the cell. Ultimately, this leads to an increase in gastric tumours [12]. In another example, it was shown that *A. aegypti* miRNA expression profile is altered when infected with *Wolbachia* [11]. The study only identified the target of aae-miR-2940, which was one of the miRNAs that showed up-regulation upon *Wolbachia* infection [11]. Despite many well-demonstrated examples such as these, there still exist large gaps in the current state of knowledge regarding the exact role of miRNAs in host-pathogen interactions, especially in invertebrates [13]. Notably, in the case of *A. aegypti* whose miRNA profile is altered in response to infection with the parasitic *w*MelPop-CLA strain of *Wolbachia* [11], very few of the gene targets of these differentially expressed miRNAs have been identified.

Using bioinformatic techniques to predict possible targets and qRT-PCR to quantify the mRNA transcript levels of those targets across various treatments, we aimed to characterize the gene target(s) of aae-miR-12 which was found to be differentially expressed in *A. aegypti* mosquitoes infected with *Wolbachia* when the previous microarray data [11] were further analyzed. Results from this study demonstrated that the *A. aegypti* genes, DNA replication licensing factor (*MCM6*) and monocarboxylate transporter (*MCT1*), were significantly down-regulated in response to *Wolbachia* infection. Further experimentation in insect cell lines concluded that this was a direct result of the up-regulation and subsequent interaction of aae-miR-12 with the targets. In addition, results suggested that aae-miR-12 is essential for *Wolbachia* replication/maintenance in the host cells.

## Results

### Bioinformatics Predicts MCM6, MCT1 and Exonuclease as Targets for aae-miR-12

NCBI BLAST searches were utilized to generate a list of homologies between the aae-miR-12 and genes within the *A. aegypti* genome. This approach produced a shortlist of 32 genes as potential targets of the miRNA. Subsequently, these genes were individually evaluated using RNAHybrid miRNA target prediction software, which produced a refined list of three most likely targets as shown in Table 1.

**Table 1:** Predicted targets of aae-miR-12.

Accession Number	Gene Function
AaeL_AAEL012546	DNA replication licensing factor MCM6
AaeL_AAEL007801	Exonuclease
AaeL_AAEL002412	Monocarboxylate transporter *MCT1*

**Figure 1:**
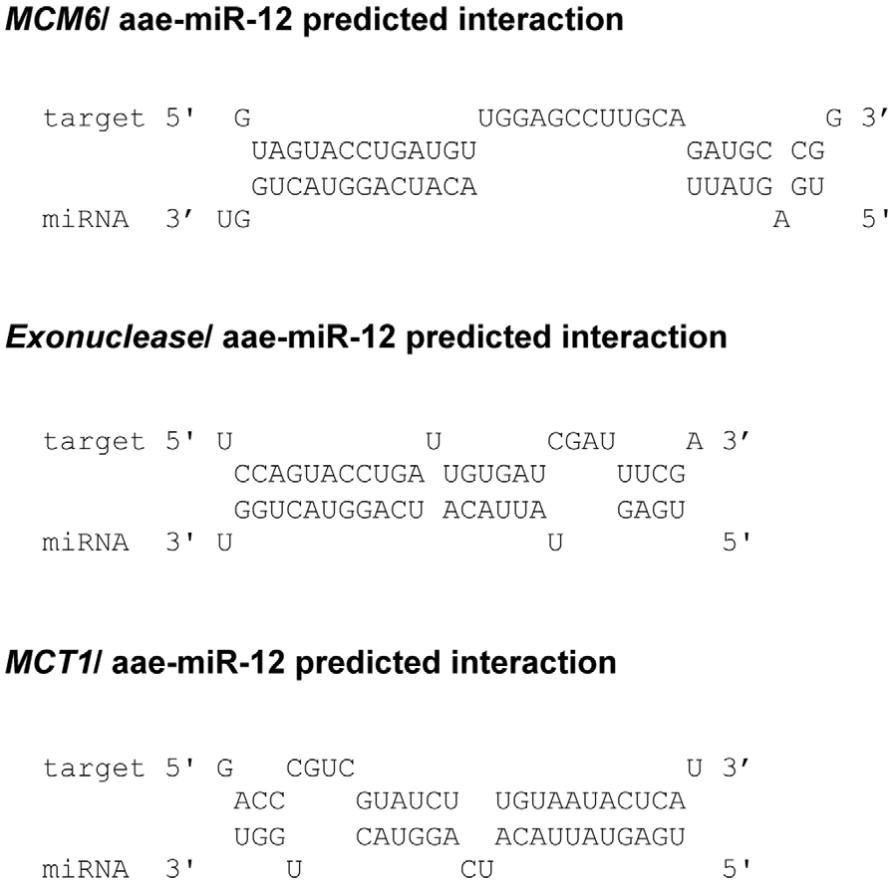
The *A. aegypti MCM6*, *Exoculease* and *MCT1* were predicted to be the best targets of aae-miR-12 with significant sequence complementarities.

These genes had predicted target sites in their open reading frames with substantial complementary sequences to the seed region or the rest of aae-miR-12 as well as a free energy of interaction which were calculated to be greatly negative; −27.6 kcal/mol for *MCM6*, −30 kcal/mol for *Exonuclease* and −27.2 kcal/mol for *MCT1* (**Figure 1**).

### *Wolbachia* Induces Suppression of MCM6 and MCT1 Genes in *A. aegypti* Cells

**Figure 2:**
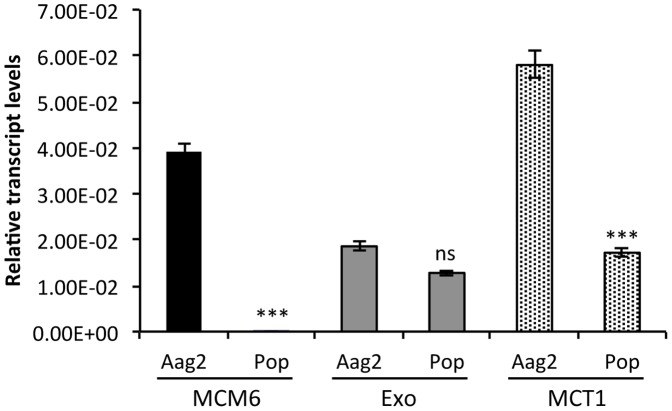
qRT-PCR analysis of predicated target genes of aae-miR-12 in aag2-*w*MelPop-CLA and uninfected Aag2 cells. The error bars indicate standard deviations of averages from two biological and three technical replicates. ***, *p*<0.001; ns, *p*>0.05.

Given the knowledge that *w*MelPop-CLA infected mosquitoes showed significant induction of aae-miR-12 when compared to uninfected mosquitoes [11], we tested the hypothesis that this miRNA has a regulatory effect on *A. aegypti* target genes predicted using bioinformatics analysis, which in turn allows the bacterium to colonize its host more effectively. Using primers specific to the *MCM6*, *MCT1* and *Exonuclease* genes, qRT-PCR assays were performed on RNA extracted from *w*MelPop-CLA infected (aag2-*w*MelPop-CLA) and uninfected Aag2 cells, a cell line derived from *A. aegypti*. Results determined a significant down-regulation of both *MCM6* and *MCT1* transcript levels in aag2-*w*MelPop-CLA cells (**Figure 2**; *p* = 0.0003). There was no significant difference in the transcript levels of *Exonuclease* (*p>*0.05) in aag2-*w*MelPop-CLA and uninfected Aag2 cells (**Figure 2**). In the case of *MCT1* and *MCM6*, the difference was concurrent with the up-regulation of aae-miR-12 in *Wolbachia*-infected (+Wol) mosquitoes based on further analysis of the microarray data [11].

### Aae-miR-12 Transfection Mimics the Effect of *Wolbachia* in Regards to MCM6 and MCT1 Regulation

**Figure 3:**
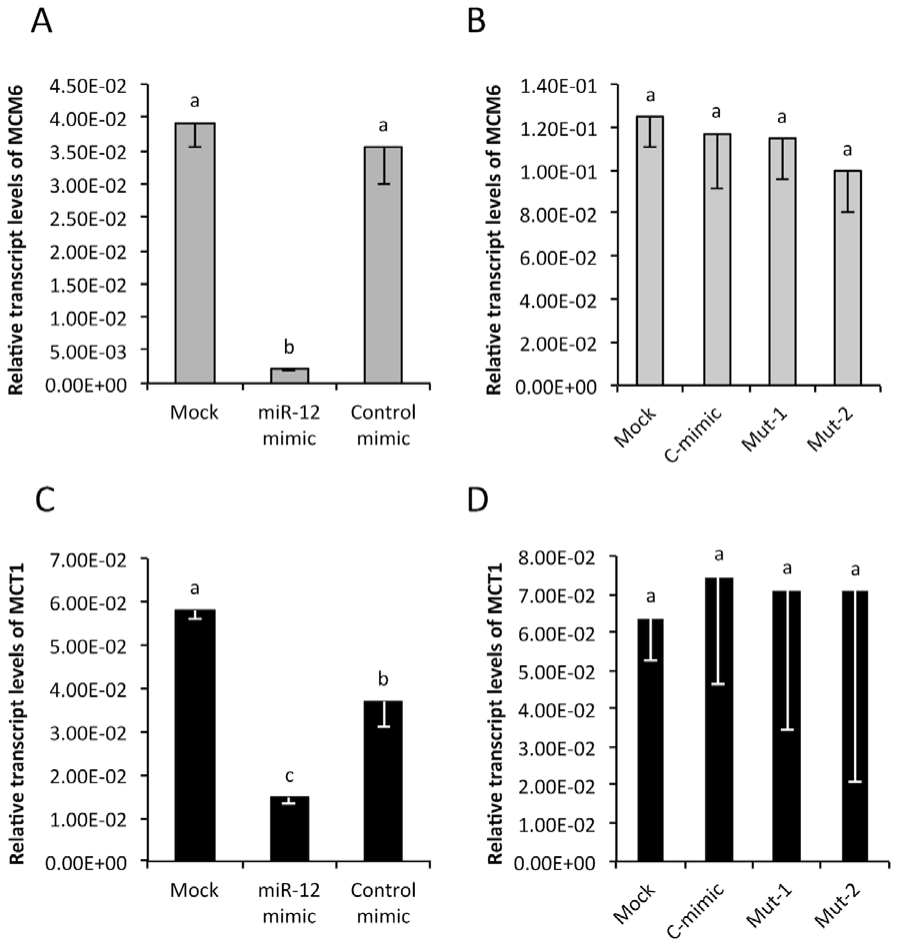
Validation of aae-miR-12 interaction with *MCT1* and *MCM6* target genes using miRNA mimics. (**A**) qRT-PCR analysis of RNA extracted from Aag2 cells mock-transfected and those transfected with the aae-miR-12 mimic or the control mimic (C-mimic). Specific primers to the *MCM6* were used. (**B**) Similar experiment as in (A) repeated with mutant mimic 1 (mut-1) and 2 (mut-2) of aae-miR-12. (**C**) qRT-PCR analysis of RNA extracted from Aag2 cells mock-transfected and those transfected with the aae-miR-12 mimic or the control mimic (C-mimic). Specific primers to the *MCT1* were used. (**D**) Similar experiment as in (C) repeated with mutant mimic 1 (mut-1) and 2 (mut-2) of aae-miR-12. The error bars in A–D indicate standard deviations of averages from two biological and three technical replicates. There are no statistical significant differences within the group with the same letter at *p*>0.05.

To confirm that down-regulation of *MCM6* and *MCT1* in *Wolbachia*-infected Aag2 cells was a result of aae-miR-12 interaction with the target genes, additional experiments were conducted transfecting Aag2 uninfected cells with the synthetic aae-miR-12 mimic. As controls, cells were mock-transfected or transfected with a control mimic, which consisted of random sequences. Aag2 cells that were transfected with the synthetic aae-miR-12 mimic showed significantly reduced transcript levels for both the *MCM6* and *MCT1* genes 72 h post-transfection (**Figure 3A, C**; *p* = 0.000224, *p* = 0.001255). The reduced transcript levels in Aag2 cells transfected with aae-miR-12 mimics were consistent with those of the aag2-*w*MelPop-CLA cells (**Figure 2**). Aag2-*w*MelPop-CLA cells transfected with the control mimic showed little observable difference to their mock-transfected counterparts (**Figure 3A, C**; *p*>0.05). To further confirm the specific interaction of aae-miR-12 with the targets, two mutant mimics of aae-miR-12, with point mutations in the regions where substantial sequence complementarity with the target exists (see Material & Methods for details), were used in similar but independent experiments. However, no significant reduction in the transcript levels of *MCM6* or *MCT1* was observed (**Figure 3B, D**; *p*>0.05).

**Figure 4:**
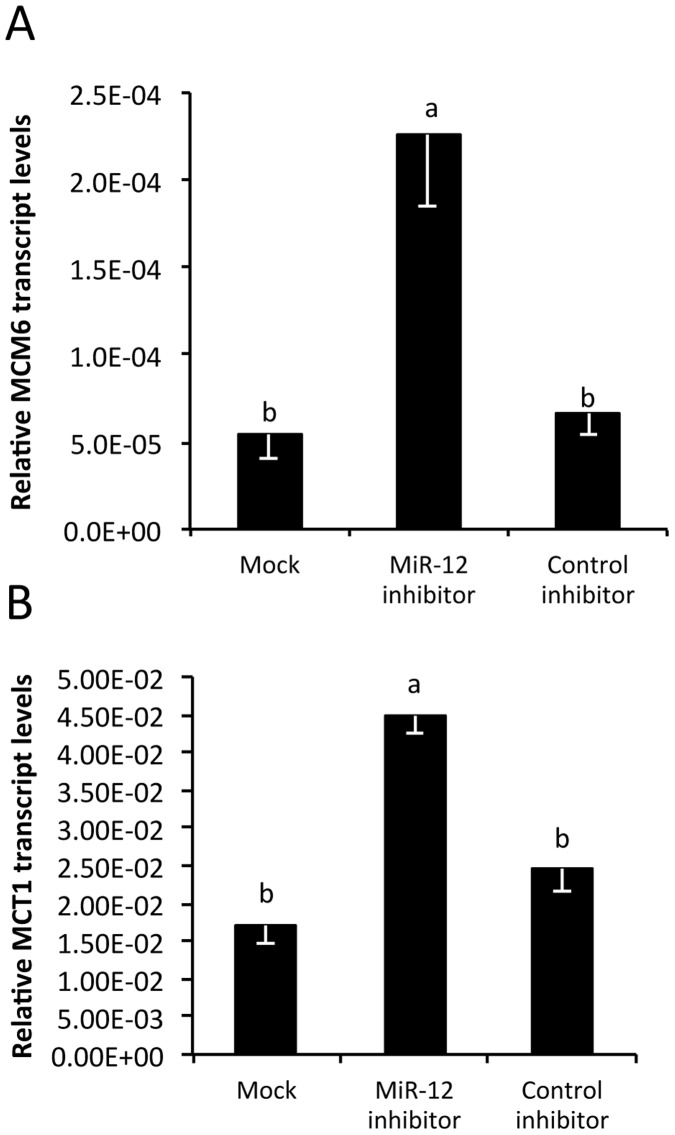
Validation of aae-miR-12 interaction with *MCT1* and *MCM6* target genes using miRNA inhibitors. qRT-PCR analysis of RNA extracted from aag2-*w*MelPop-CLA cells mock-transfected, transfected with aae-miR-12 inhibitor and control inhibitor and analysed with specific primers to (**A**) *MCM6* and (**B**) *MCT1*. The error bars indicate standard deviations of averages from two biological and three technical replicates. There are no statistical significant differences within the group with the same letter at *p*>0.05.

Subsequently, we proceeded to test the hypothesis that if indeed aae-miR-12 is responsible for the down-regulation of *MCM6* and *MCT1* then transfection of a synthetic aae-miR-12 inhibitor would rescue the mRNA transcript levels of these genes in *Wolbachia*-infected cells. Results indicated exactly this in the case of aag2-*w*MelPop-CLA cells which had previously been shown to have significantly suppressed transcript levels of both *MCM6* and *MCT1* (**Figure 4A, B**; *p* = 0.000249, *p* = 0.000324). In these cells, transfection with the synthetic aae-miR-12 inhibitor decisively up-regulated the *MCM6* and *MCT1* transcripts. The control inhibitor had no effect on the transcript levels of either of the genes (**Figure 4A, B**; *p*>0.05).

**Figure 5:**
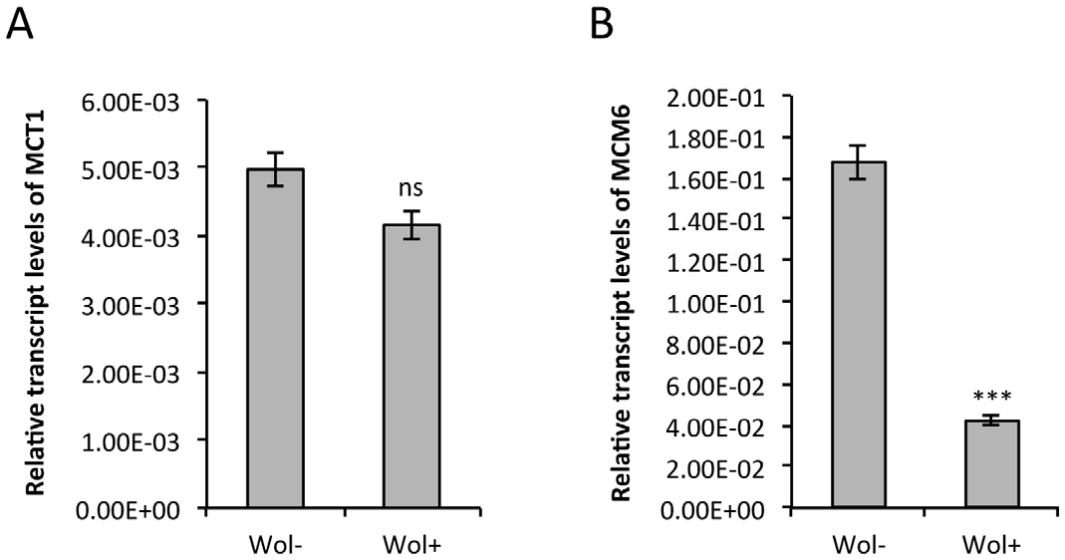
In vivo effect of *Wolbachia* on *MCM6* and *MCT1* transcript levels. qRT-PCR conducted on total RNA extracted from whole female mosquitoes. Specific primers were used for (**A**) *MCM6* and (**B**) *MCT1* to determine transcript levels in mosquitoes infected with *Wolbachia* (Wol+) as well as those not infected (Wol−). The error bars indicate the standard deviation in the averages of 3 biological and 3 technical replicates. The cumulative tissue from ten whole mosquitoes (4 days old) was used for each biological replicate. ***, *p*<0.001; ns, not significant *p*>0.05.

In order to determine whether the results achieved in vitro were consistent with similar processes in vivo, the transcript levels of *MCM6* and *MCT1* in total RNA extracted from whole female mosquitoes (4 days after emergence) were determined. The results from these experiments revealed that there was significantly lower transcript levels of *MCM6* (**Figure 5A**; *p* = 0.000209), and reduced levels of *MCT1* (**Figure 5B**) in+Wol mosquitoes compared to uninfected –Wol mosquitoes; although the difference in the case of MCT1 was not statistically significant (*p* = 0.109) as in the cell line.

### Aae-miR-12 Positively Interacts with MCM6 and MCT1 Target Sites in Sf9 Cells

**Figure 6:**
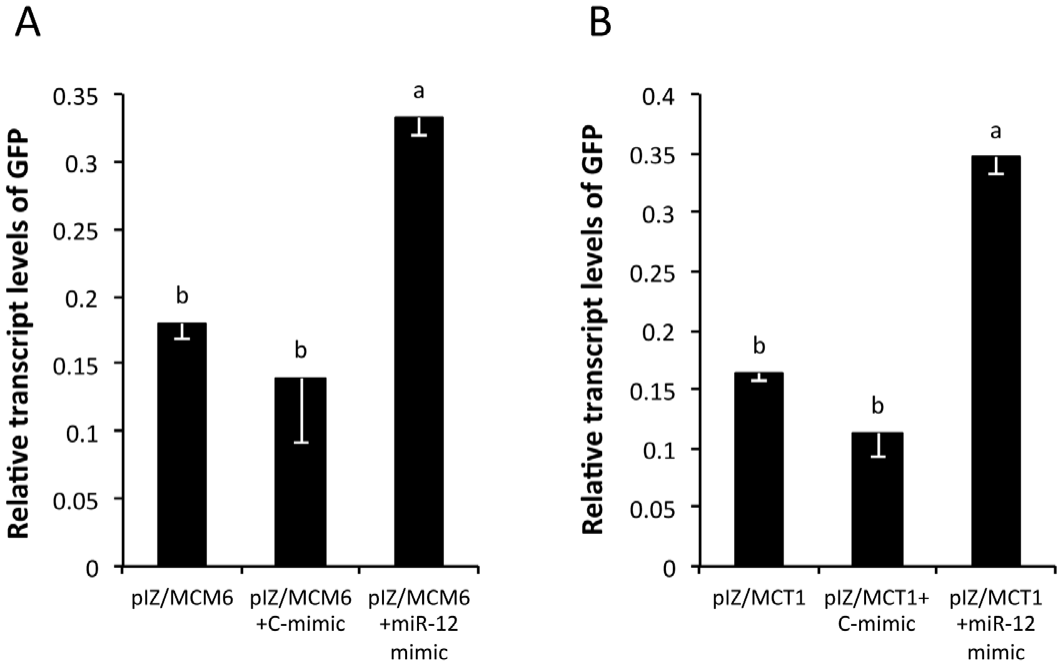
qRT-PCR analysis of RNA extracted from Sf9 cells co-transfected with either (A) pIZ-GFP-*MCM6* (pIZ/MCM6) or (B) pIZ-GFP-*MCT1* (pIZ/MCT1) constructs and control mimic (C-mimic) or aae-miR-12 mimic. The error bars indicate standard deviations of averages from two biological and three technical replicates. There are no statistical significant differences within the group with the same letter at *p*>0.05.

To test the interaction of aae-miR-12 with ORF target sites of both the *MCT1* and *MCM6* genes, both target sites were cloned downstream of a GFP reporter gene in the commercially available pIZ/V5 vector. These were subsequently co-transfected into Sf9 cells (derived from *Spodoptera frugiperda*) together with a control mimic or aae-miR-12 mimic. The expression of GFP in Sf9 cells using the vector is optimal and also the cell line provides an independent system to test the miRNA-target interaction. Using primers specific to the GFP reporter sequence, qRT-PCR analyses were conducted to assess the effect of miRNA-mRNA interaction on the *GFP* transcript levels. It was determined that whilst very little difference could be observed in the levels of GFP reporter transcripts between the mock and the control mimic treatments (*p*>0.05), there were significantly higher levels of *GFP* transcripts in those cells transfected with the aae-miR-12 mimic (**Figure 6**; *p* = 0.003, *p* = 0.006). This was the case for both the pIZ-GFP-*MCT1* (**Figure 6A**) and pIZ-GFP-*MCM6* (**Figure 6B**) constructs.

### Inhibition of aae-miR-12 Reduces the *Wolbachia* Density in Mosquito Cell Line

**Figure 7:**
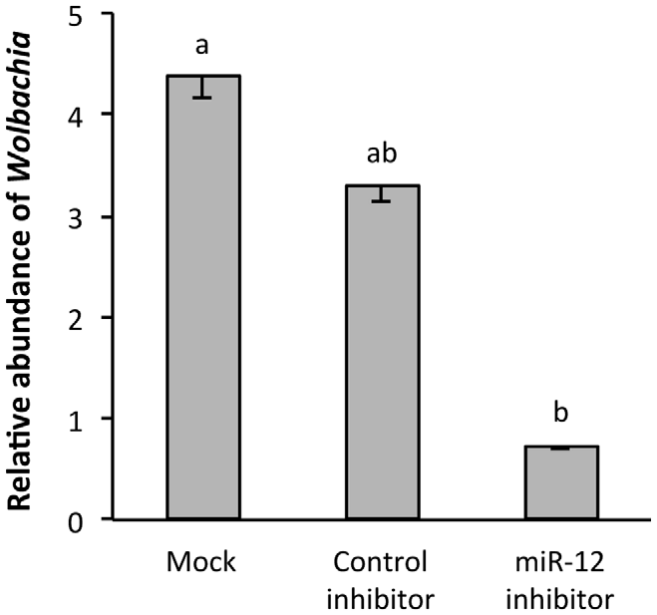
Inhibition of aae-miR-12 affects *Wolbachia* density. qPCR analysis of DNA isolated from aag2-*w*MelPop-CLA cells mock-transfected, transfected with the control inhibitor or aae-miR-12 inhibitor. The error bars indicate standard deviations of averages from two biological and three technical replicates. There are no statistical significant differences within the group with the same letter at *p*>0.05.

Given that *Wolbachia* infection regulates the expression of aae-miR-12 in mosquitoes, we hypothesised that this miRNA has a crucial role in mediating the presence of cellular proteins, which serve to further the bacterium’s ability to persist in the cells. Equal numbers of aag2-*w*MelPop-CLA cells were mock-transfected, transfected with the synthetic aae-miR-12 inhibitor or the control inhibitor. Using the primers specific to *wsp* gene (normally used to quantify *Wolbachia* density), a qPCR was undertaken on genomic DNA extracted from each of the treatments 72 h after the transfection. The results indicated that as opposed to the mock and the control inhibitor treatments, aae-miR-12 inhibitor substantially reduced the density of *Wolbachia* in the host cells (**Figure 7**; *p* = 0.0049).

## Discussion

Although many efforts have been dedicated to understanding the biology of *Wolbachia*, there still exists a plethora of unexplored mechanisms underlying its interactions with other organisms, through which it appears to be manipulating its host’s environment in an attempt to insure the bacterium’s survival. The findings of Hussain et al. (2011) [11] have shed light on what appears to be one of the molecular mechanisms by which *Wolbachia* mediates changes in the host *A. aegypti.* The functional analyses carried out in the report demonstrated that mosquito cellular miRNAs are differentially expressed as a result of *Wolbachia* infection, a finding consistent with current literature, which describes similar phenomena in other host-pathogen interactions across Eukarya [2], [14], [15]. One such example in humans is infection with *H. pylori,* which induces miR-155 in T cells [12]. Hussain et al. (2011) went on to further illustrate the specific role of one of these differentially expressed miRNAs, aae-miR-2940, in *Wolbachia* in regulating a metalloprotease cellular protein, which is imperative in facilitating *Wolbachia*’s colonization of its mosquito host [11].

In this study, the regulatory role of another differentially expressed mosquito miRNA, aae-miR-12, which is up-regulated by *Wolbachia*, is described. Based on bioinformatics predictions, we investigated the miRNA-mRNA interactions of aae-miR-12 and three characterized mosquito target genes. Quantitative-Real Time PCR analyses of levels of mRNA transcripts revealed that both the *MCT1* and *MCM6* genes were suppressed by this miRNA in vitro and in vivo. However, there was no effect on *Exonuclease* transcript levels; therefore, further studies concentrated on *MCT1* and *MCM6* target genes. In addition, further functional analyses demonstrated the crucial role aae-miR-12 plays in *Wolbachia*’s fitness in the mosquito cell line tested.

Confirmation of the interaction of aae-miR-12 with the predicted targets was first attempted through the use of a synthetic aae-miR-12 mature miRNA mimic in cells not infected with *Wolbachia* to see if aae-miR-12 alone could mimic *Wolbachia*’s effect on *MCT1* and *MCM6* genes. The results indicated that this was indeed the case. This finding was further scrutinized by testing the logical prediction generated by the experiment that if indeed artificially inducing aae-miR-12 could mimic *Wolbachia*’s down-regulation of *MCT1* and *MCM6* genes, then conversely inhibiting aae-miR-12 in *Wolbachia-*infected cells should reverse the down-regulation of these genes. Upon transfection of *Wolbachia*-infected cells with the miRNA inhibitor of aae-miR-12, it was discovered that this treatment could in fact prevent *Wolbachia* from down-regulating *MCT1* and *MCM6,* providing further evidence in support of the hypothesis that aae-miR-12 interacts with the respective predicted target sites in both of the target genes. Surprisingly, when GFP was used as a reporter gene in the pIZ vector, downstream of which target sites for both *MCT1* and *MCM6* genes, respectively, were cloned and then transfected into Sf9 cells, the synthetic aae-miR-12 mimic had the effect of significantly increasing GFP transcript levels rather than decreasing them. This finding is directly opposite to the trends observed in *A. aegypti* cells. Upon further inspection of the literature however, it appears that this phenomenon is not undescribed. For example, Callis et al. (2009) also showed that miRNA can have opposing effects on transcript levels depending on the miRNA-reporter binding context [16]. In this instance, the context of interaction which is downstream of GFP differs from the interaction in target gene mRNAs in that the target sites for *MCT1* and *MCM6* genes are in the coding region. In this experiment however, in the GFP construct the target sites become localized to what effectively becomes the 3′UTR of the GFP transcript, thus the nature of the interaction is altered and produced a different outcome. These findings may lend themselves to the hypothesis that aae-miR-12 is interacting with target sites in both *MCT1* and *MCM6,* but the fact that the nature of the interaction differs depending on the cellular circumstances highlights the detail that the precise method of transcript regulation is still elusive. As miRNAs can interact with their targets in many ways, this represents an interesting avenue for further research into these specific interactions.

Considering that cell lines represent in vitro experimental systems and may not necessarily be representative of in vivo processes affecting overall mosquito’s physiology, we undertook quantitative experimentation on RNA extracted from whole mosquitoes to determine the overall expression of *MCM6* and *MCT1* in vivo in+Wol and –Wol mosquitoes. Results of these experiments showed the same pattern of expression for our predicted target genes across the treatments as we observed in cell lines. In mosquitoes infected with *Wolbachia*, we observed significantly lower levels of *MCM6* transcripts than in uninfected mosquitoes and whilst we saw a similar reduction in the case of *MCT1,* the reduction was not statistically significant. This is likely due to the fact that *MCT1* is not expressed in all mosquito tissues, which would imply that the transcript reduction effect would be diluted in a total RNA extraction. This is not the case in a clonal cell culture in which all cells may express the gene.

We derived the hypothesis that if indeed *Wolbachia* had a role to play in regulating cellular proteins through induction of aae-miR-12, then inhibiting this process would somehow have a detrimental effect on the ability of *Wolbachia* to persist in the host cell. In order to test this hypothesis, we transfected Aag2 cells infected with *Wolbachia* with aae-miR-12 inhibitors and measured the effect this had on *Wolbachia* density using the *wsp* gene as an indicator. qRT-PCR results showed that whilst a control inhibitor with scrambled sequence had little effect on the number of detectable copies on the *wsp* gene in *Wolbachia*-infected cells, transfection of the aae-miR-12 inhibitor significantly reduced *Wolbachia* density. This suggests that aae-miR-12 plays a critical role in modifying the cellular environment of the endosymbiont’s host in order to increase that environment’s habitability for *Wolbachia* by changing the levels of *MCM6* and *MCT1* proteins within the cell.

The *Drosophila melanogaster* homologue of MCM6 has been demonstrated to play a critical role in chorion gene amplification as well as genomic replication [17]. This function in coding a DNA replication licensing factor has been well documented and attributed to MCM6 across a broad range of eukaryotes [18]. This is a strong indication that this gene is well conserved across eukaryotes. Also in *Drosophila*, *MCT1* encodes a monocarboxylate transporter which serves to provide metabolic fuel such as lactate and pyruvate to cells [19]. Exactly what function or specific benefit may be conferred to *Wolbachia* by suppressing *MCM6* and *MCT1* genes in host cells is as of yet unclear and requires further investigations.

In conclusion, we have demonstrated the importance of the cellular miRNA aae-miR-12 in *Wolbachia* colonization of *A. aegypti* Aag2 cells. Our results demonstrated that inhibition of this miRNA drastically reduces *Wolbachia*’s persistence in host cells. Furthermore, we have identified potential targets of this miRNA through bioinformatics techniques. These targets, *MCM6* and *MCT1* genes, were functionally validated and demonstrated to be under the regulative control of aae-miR-12 both in vitro and in vivo. The findings demonstrated here merit further study into the precise role the proteins encoded by these target genes play in mediating host-pathogen interactions.

## Materials and Methods

### Maintaining Mosquitoes and Cell Lines

Previously, McMeniman et al. (2008) [4] had generated mosquitoes and cell lines infected with *w*MelPop-CLA strain of *Wolbachia* and also those cured with tetracycline, which were utilized in the experiments. For those cells infected with *w*MelPop-CLA, the procedure of infection is described by Frentiu et al. (2010) [20]. PCR assays were conducted to ensure stable infection continued throughout experimentation. Aag2 cells derived from *A. aegypti* were cultured in Schneider’s medium (Life Technologies) supplemented with 10% fetal bovine serum (FBS).

### miRNA Target Predictions

The *A. aegypti* genome was screened for potential targets bearing homology to aae-miR-12 using NCBI BLAST. A list of 32 potential targets was generated in this way. These candidates were then analysed with the RNAHybrid and RNA22target prediction software to confirm their potential interaction with aae-miR-12 as well as all possible binding sites for the miRNA. From this information, a refined target list of 3 different genes was compiled (Table 1).

### Inhibition and Mimic of aae-miR-12 in Mosquito Cell Line

The aae-miR-12 inhibitor (reverse complement RNA oligo) and mimic (UGAGUAUUACAUCAGGUACUGGU) were synthesized by Genepharma along with control “scramble” inhibitor (UCUACUCUUUCUAGGAGGUUGUGA) and mimic (UUCUCCGAACGUGUCACGUTT). In addition, two mutant mimics with two nucleotide mutations in the complementary regions with the targets were synthesized and used in transfections (mutant mimic-1 UGAGUAUUACAUCAAAUACUGGU and mutant mimic 2 UGAACAUUACAUCAGGUACUGGU; mutated residues are underlined). Transfection of inhibitors and mimics was undertaken using 2 µg of RNA. Cellfectin was used as the transfection reagent according to the manufacturer’s instructions (Invitrogen). Total RNA/DNA extraction from these cells was performed 72 hours post-transfection.

### qPCR Analyses

Total RNA was extracted from cells using TRI Reagent according to the manufacturer’s instructions (Molecular Research Centre). The concentration of RNA was measured using nanodrop. Dilutions of 100 ng/µl RNA were then produced for further experimentation. Gene-specific reverse primers were used in conjunction with reverse transcriptase Superscript II (Invitrogen) to generate cDNA to subsequently be amplified in qPCR reactions. The temperature conditions for the 1^st^ strand synthesis began with annealing the primers to the RNA template at 65°C for 5 min followed by 1 h at 50°C.

Both forward and reverse primers specific to the target gene being tested were used for qPCR reactions. Each qPCR reaction was performed in triplicates in two biological replicates. qPCR primers used for *MCM6* were MCM6-For 5′-GGAGCCTTGCAGATGCCGGG-3′ and MCM6-Rev 5′-CGCTTGCACCGAACAGGCCA-3′, and for *MCT1* were MCT1-For 5′-CACCCGTGCCTCTTACCCCG-3′ and MCT1-Rev 5′-AGCCCCATCCACCATCGGGT-3′. The qPCR cycling conditions were 94°C for 3 min, 94°C for 15 sec, 60°C for 40 sec and 72°C for 45 sec, repeated for 36 cycles. For qPCR reactions using genomic DNA as the template a 2 min melt step was added and the melt curve was analysed to ensure amplification was a uniform product. Melting curves were analyzed after each run to check the specificity of amplification. The relative ratio of *MCM6* and *MCT1* mRNAs to cellular RNA (*RPS17* used for normalizing data) was determined using the specific primers given above and *RPS17* specific primers (rsp17-For 5′-CACTCCGAGGTCCGTGGTAT-3′ and rsp17-Rev 5′-GGACACTTCGGGCACGTAGT-3′).

DNA extraction was performed using a homogenization mixture as per Invitrogen instructions, and primers specific to the *Wolbachia* housekeeping gene *Wsp* were wsp-For 5′-GTCCAATATSTGATGACGAAAC -3′ and wsp-Rev 5′- CTGCACCAACAGGCCTATAAA -3′. All qRT-PCR data was subjected to statistical analyses using ANOVA and *t*-tests followed by Tukey’s test where relevant.

### Cloning Targets Under GFP in pIZ

Fragments approximately 250 bp long from both *MCM6* and *MCT1* containing the target sequences of aae-miR-12 were amplified from total *A. aegypti* RNA using primers that were designed to amplify these fragments with specific restriction sites *Xba*I and *Sac*II (New England Biosciences) for cloning into the pIZ vector. These primers were qMCM6-For 5′-GTCTAGAAGTGATTCTTCGGGCTGAAA-3′, qMCM6-Rev 5′-GCCGCGGGCCAGGAAAGCCATCTTGTA-3′ for *MCM6* and qMCT1 For 5′- GTCTAGATCTGTCGCTGAGATCGATTG-3′, qMCT1-Rev 5′-GCCGCGG AGATTGCGTATGCGGGTAAC-3′ for *MCT1.* The fragments containing the restriction sites were then purified from agarose gel using QIAquick Gel Extraction Kit (QIAGEN) and ligated into the pIZ vector (Invitrogen) containing GFP. Vectors which could be digested with *Eco*RI and *Sac*II to liberate fragments of the predicted size were sequenced to confirm the identity of the cloned fragments. Transfection of these plasmids into insect cells was performed using 2 µg of the plasmid per transfection. As a control, cells were transfected with pIZ vector containing only GFP. Cellfectin was used as the transfection reagent according to the manufacturer’s instructions (Invitrogen). Primers specific to the GFP sequence (GFP-For 5′-CCCAAGCTTCGCCACCATGGTGAGCAA-3′and (GFP-Rev 5′- CGGGGTACCCTTGTACAGCTCGTCCATGC-3′) were used for the subsequent qRT-PCR 72h after transfection. Data were analysed as above using actin as the normalizing gene. The primers used for actin were actin-For 5′-ATGGAGAAGATCTTGCAC-3′ and actin-Rev 5′-GGAGCCTCCGTGAGCAGC-3′.
